# Back to the Basics: A COVID‐19 Surveillance Program Within a Local School District

**DOI:** 10.1111/josh.13149

**Published:** 2022-02-23

**Authors:** Prakash R. Ganesh, Ross May, Mitch Dandurand, Jeffrey Graham, Johnie Rose, Heidi Gullett, Dave Covell, Kurt C. Stange

**Affiliations:** ^1^ Department of Family Medicine and Community Health, University Hospitals Cleveland Medical Center, Cleveland, OH; Center for Community Health Integration, Case Western Reserve University School of Medicine, 11000 Cedar Avenue, Suite 402 Cleveland OH 44106‐7136 USA; ^2^ Lorain City School District, 2601 Pole Avenue Lorain OH 44052 USA; ^3^ Lorain County Public Health, 9880 South Murray Ridge Road Elyria OH 44035 USA; ^4^ Lorain City School District, 2601 Pole Avenue Lorain OH 44052 USA; ^5^ Center for Community Health Integration, Case Western Reserve University School of Medicine, Cleveland, OH, 11000 Cedar Avenue, Suite 402; Department of Family Medicine and Community Health, University Hospitals Cleveland Medical Center Cleveland OH 44106‐7136 USA; ^6^ Center for Community Health Integration, Case Western Reserve University School of Medicine, Cleveland, OH, 11000 Cedar Avenue, Suite 402; Department of Family Medicine and Community Health, University Hospitals Cleveland Medical Center Cleveland OH 44106‐7136 USA; ^7^ Lorain County Public Health, 9880 South Murray Ridge Road Elyria OH 44035 USA; ^8^ Center for Community Health Integration, Case Western Reserve University School of Medicine, Cleveland, OH, 11000 Cedar Avenue, Suite 402; Department of Family Medicine and Community Health, University Hospitals Cleveland Medical Center Cleveland OH 44106‐7136 USA

**Keywords:** COVID‐19, surveillance, schools, transmission, mitigation

## Abstract

**BACKGROUND:**

A school district in Northern Ohio implemented a COVID‐19 surveillance program from January 4 to May 21, 2021, as in‐person school and extracurricular activities resumed.

**METHODS:**

Among 560 staff members and >6300 students, random weekly testing was performed on 563 students and weekly for 204 students participating in extracurricular activities, and 553 staff.

**RESULTS:**

Cases of COVID‐19 were identified among 26 staff members and 23 students. Most of those infected were participating in extracurricular activities (14/23) and in the age range of 14‐18. Percent positivity was low (range 0.2‐2.4%) throughout the school surveillance program despite significant changes in positivity rate (2.8‐19.8%).

**CONCLUSION:**

This demonstrates that in a setting employing basic yet consistent mitigation strategies, there is low transmission among young children and adolescents as they return to in‐person classes and activities. Maintaining layered prevention strategies implemented and sustained with fidelity can substantially limit transmission within schools.

Early in the pandemic, schools throughout the country closed in‐person instruction to prevent transmission of the novel SARS‐CoV‐2 virus. This approach has had a substantial effect on the 50,000,000 students and 5,000,000 adults within the US K‐12 public school system^1^ and has the potential to continue adversely affecting students' academic progress, mental health, and essential services.^2^

To date, there has been little evidence that school environments contribute to increased community transmission.^3^ In addition, within‐school transmissions appear to be rare,^1^ and COVID‐19 incidence is often lower in schools than in the community.^4^ However, there have been reported outbreaks within weeks of school reopening, attributed to crowded classrooms with insufficient physical distancing, exceptions for face mask use, and continuous air conditioning in closed rooms.^5^ In addition, those participating in extracurricular activities may be at increased risk of SARS‐CoV‐2 transmission.^3^

As schools attempt to safely re‐open to in‐person instruction, minimal and widely varying guidance and limited resources have made it difficult for schools to implement effective testing approaches. Therefore, we aimed to elucidate transmission patterns among staff and students during the re‐opening of a large public school system, to monitor the incidence of COVID‐19, to assess the effectiveness of mitigation strategies, isolation and quarantine measures, and to inform future policy regarding in‐person instruction.

## METHODS

After 10 months of exclusively virtual instruction, the Lorain City School District (LCSD), partnering with Lorain County Public Health (LCPH) and Mercy Health, a regional health care system, implemented COVID‐19 polymerase chain reaction (PCR) surveillance testing with in‐person re‐opening.

LCSD is located in Northeast Ohio and serves more than 6300 students in pre‐Kindergarten to 12th grade within 14 buildings. The surveillance program was conducted from January 4, 2021 to May 21, 2021, with the assistance of Mercy Health.

### Participants

Candidates for surveillance testing included enrolled students and staff returning to in‐person classes or extracurricular activities that would not allow for the maintenance of proper mitigation strategies within all schools in the district. Student and staff sociodemographics are described in Table 1.

**Table 1:** Demographic and Surveillance Testing Data for Students and Staff*

Students	General, n (%)	Positive, n (%)	Extracurricular, n (%)	Positive, n (%)
Age, mean (SD)	9.75 (3.30)	12.1 (3.0)	15.9 (1.4)	16.1 (1.2)
Gender				
Female	266 (47.8)	2 (22.2)	87 (42.7)	3 (21.4)
Male	290 (52.2)	7 (77.8)	117 (57.4)	11 (78.6)
Race				
Asian	1 (0.2)	0	0 (0.0)	0 (0.0)
African American	135 (24.3)	2 (22.2)	81(39.7)	8 (57.1)
Hispanic/Latino	246 (44.2)	6 (66.7)	76 (37.3)	4 (28.6)
Unknown	1 (0.2)	0	1 (0.5)	0 (0.0)
Multiracial	42 (7.6)	0	16 (7.8)	1 (7.1)
Caucasian	131 (23.6)	1 (11.1)	30 (14.7)	1 (7.1)
Grade				^†^
Preschool	36 (6.4)	0	0	0
Kindergarten	60 (10.7)	0	0	0
1st	58 (10.3)	0	0	0
2nd	70 (12.4)	1 (11.1)	0	0
3rd	60 (10.7)	1 (11.1)	0	0
4th	52 (9.2)	1 (11.1)	0	0
5th	53 (9.4)	1 (11.1)	0	0
6th	34 (6.0)	0	1 (0.5)	0
7th	39 (6.9)	1 (11.1)	4 (2.0)	0
8th	55 (9.8)	3 (33.3)	12 (5.9)	0
9th	18 (3.2)	0	66 (32.4)	2 (14.3)
10th	18 (3.2)	0	35 (17.2)	8 (57.1)
11th	8 (1.4)	1 (11.1)	45 (22.0)	2 (14.3)
12th	2 (0.4)	0 (0.0)	41 (20.1)	2 (14.3)
Disability^‡^				
Yes	118 (21.0)	4 (44.4)	16 (7.8)	2 (14.3)
No	445 (79.0)	5 (55.6)	188 (92.2)	12 (85.7)

Staff	Negative, n (%)	Positive, n (%)	Total, n (%)	p‐value
Age mean (SD)	47.0 (11.9)	47.3 (13.5)	47.0 (12.0)	
Gender				
Female	384 (79.8)	18 (69.2)	402 (79.3)	.214
Male	97 (20.2)	8 (30.8)	105 (20.7)	
Race				
Asian	5 (1.0)	0 (0.0)	5 (1.0)	.970
African American	43 (8.9)	3 (11.5)	46 (9.1)	
Hispanic/Latino	88 (18.3)	5 (19.2)	93 (18.3)	
Caucasian	345 (71.7)	18 (69.2)	363 (71.6)	
Job role				
Admin assistant	28 (5.3)	3 (10.7)	31 (5.6)	.334
Central office	22 (4.2)	1 (3.6)	23 (4.2)	
Coach	2 (0.4)	0 (0.0)	2 (0.4)	
Counselor	7 (1.3)	0 (0.0)	7 (1.3)	
Custodian/Cleaner	24 (4.6)	1 (3.6)	25 (4.5)	
Finance	7 (1.3)	0 (0.0)	7 (1.3)	
Food service	14 (2.7)	0 (0.0)	14 (2.5)	
Head Start	6 (1.1)	2 (7.1)	8 (1.5)	
Health Pro	5 (1.0)	0 (0.0)	5 (0.9)	
Occupational Ther	2 (0.4)	0 (0.0)	2 (0.4)	
Paraprofessional	51 (9.7)	3 (10.7)	54 (9.8)	
Principal	21 (4.0)	0 (0.0)	21 (3.8)	
Psychologist	5 (1.0)	0 (0.0)	5 (0.9)	
Safety officer	7 (1.3)	2 (7.1)	9 (1.6)	
Social worker	6 (1.1)	0 (0.0)	6 (1.1)	
Substitute	10 (1.9)	0 (0.0)	10 (1.8)	
Teacher	308 (58.7)	16 (57.1)	324 (58.6)	

* p‐Values were calculated for comparisons between positives vs the corresponding cohort and between positives between both testing groups. Total of 9 and 14 positives in the general and extracurricular cohorts, respectively.

^†^ p‐Value <.05.

^‡^ Disabilities included autism, intellectual disability, deafness, blindness, developmental delay, orthopedic impairments, visual impairments, learning disabilities, and traumatic brain injury. The majority of disability is due to specific learning disabilities (46.9%).

### Procedure

When classes resumed, anterior nares nasal swabs were collected from consenting staff and students. After baseline testing, weekly swabs were obtained in randomly selected students from the general population by using a random number generator. If someone had previously tested positive, they were excluded from repeat testing. Testing was also performed weekly in all consenting students participating in high‐risk extracurricular activities where COVID‐19 mitigation strategies were not in place.

Weekly testing was performed for 563 students from the general population, 204 students participating in extracurricular activities, and 553 staff. Consent forms for testing were sent through internal school mail for staff and through emails and text messages to students' homes. Parental consent was obtained for all participating students. A total of 56% of staff participated, whereas 14% of the total student population and 29% of students in in‐person learning participated.

PCR testing was returned within 24‐48 hours and all positive results were reported through the Ohio Disease Reporting System which provided case notifications to LCPH. LCPH trained school staff to perform contact tracing through a centralized district team that met daily to review surveillance test results and cases who were tested elsewhere. The district team consisted of 1 central office director, the district nurse, and 6 administrative assistants. Each district contact tracer was assigned to support specific schools. The team met daily to review new cases from overnight case reporting. The district contact tracer then contacted the school to verify the new cases and to initiate contact tracing, notify the family of the positive case, or confirm information from the family for the positive case. Each school building also had a contact tracer: the principal, assistant principal, or secretary. The building contact tracer determined if close contacts existed and then entered them into the school system. As soon as the building contact tracing was complete, the district tracer notified the families of any close contacts who would be placed in quarantine by the health department. The process applied for both staff and students. LCPH assisted with isolation orders for those with positive results. Negative results were reported to a website and staff and students' families were notified via phone and website. In addition, there were weekly meetings of the surveillance program implementation partners—the Lorain County Health Department, Mercy Health, and Case Western Reserve University School of Medicine.

School administrators, research staff, and local public health officials used newsletters, videos, town hall meetings, and local media to inform students, their families, and staff members about the rationale for the surveillance program.

The district consistently maintained the following mitigation strategies for the duration of the study: cohorted classes with reduced capacity, universal masks except when eating, a late morning snack and packed lunches for all students to take home, maintenance of 6 feet of distances between all desks and seated areas, screening for symptoms, and sanitation of all common surfaces. These mitigation strategies were not possible in extracurriculars.

This study protocol was approved by the Case Western Reserve University Institutional Review Board.

### Data Analysis

Statistical analysis was carried out using Stata 14.2 (Stata Corporation, College Station, TX). Continuous variables were reported using mean and standard deviation. Categorical variables were reported as frequencies and percentages. Bivariate analyses were performed using Welch's *t*‐test for continuous variables and Fisher's exact or Pearson chi‐square for categorical variables. All p‐values reported are 2‐sided and p < .05 was considered significant.

## RESULTS

Students tested were ages 3 to 18, 46.5% (353/760) female, 42.4% (322/760) Hispanic/Latino, 28.4% (216/760) African American, and 21.2% (161/760) Caucasian. Those in extracurricular activities were in grades 6‐12. There were no significant differences between demographics of students participating in extracurricular activities and those in the general population except for age. The majority of tested staff were female (79.3%) and 71.6% Caucasian, 18.3% Hispanic/Latino, and 9.1% African American (Table 1).

Nine positive cases were identified through surveillance testing in the general student population and 14 cases among those involved in extracurricular activities, all 14‐18 years of age. There were no significant differences between those who tested positive except for age and grade. All positive results recorded in extracurricular activities were among high school students. There were no significant findings for students with disabilities.

Twenty‐six cases were identified through staff surveillance testing. There were no significant demographic differences between those who tested positive and negative.

During the period of the surveillance program, an additional 58 students and 11 staff were identified with positive COVID‐19 tests outside of our surveillance testing. These additional cases were not included in the analysis comparing both groups due to missing demographic information.

The percent positivity was fairly constant (range 0.2‐2.4%) throughout the school surveillance program despite significant changes in community positivity rate (2.8‐19.8%). This included both those who were tested within the school surveillance program and those tested in the community (Figure S1).

No adverse effects were reported from anterior nasal samples collected for all age groups. Other potential social or emotional adverse events that could have arisen from isolation or quarantine were not systematically collected or reported.

## DISCUSSION

This study adds to emerging literature indicating that regardless of COVID‐19 transmission rates in the community, schools, and children have generally lower rates of COVID‐19 infection to the initial circulating SARS‐CoV‐2 virus.^6^ This analysis demonstrates that in a setting employing basic yet consistent mitigation strategies, low transmission among young children and adolescents appears to make schools fairly safe as they return to in‐person classes and activities. Schools should evaluate the level of community transmission and local vaccination rates as they assess the risk of transmission within schools.

Maintaining layered prevention strategies implemented and sustained with fidelity can substantially limit transmission within schools.^6^ From November 2020 to March 2021, the Utah Department of Health in collaboration with local public health departments and school partners provided training and rapid antigen test kits to school staff members to implement a surveillance system to initiate a Test to Play and Test to Stay program which would allow participation in extracurricular activities and/or continued in‐person instruction as an alternative to remote instruction. The surveillance program allowed for the safe completion of over 95% of extracurricular instruction and saved in‐person instruction student‐days and concluded that a school‐based testing program was an essential prevention strategy to identify infections in schools and sustain in‐person activities.^7^

It should be noted that comparing the positivity rate in the school surveillance program may not be equivalent and would be expected to be lower than community positivity rates because the school program surveilles asymptomatic individuals compared to symptomatic individuals in the community. The main purpose of the comparison between the school and community groups was to indicate that the rates of infection in the community were not driving school infections.

Although the costs of conducting PCR testing were notable ($85/test; 8167 tests; total $694,195 USD), it did allow for monitoring of mitigation strategies. PCR testing for COVID‐19 may not be sustainable everywhere and vaccination rates may impact testing necessity. However, our surveillance system showed that COVID‐19 transmission remained low despite community levels of infection with strict adherence to mitigation efforts. In addition, a 16‐state consortium has since been developed to provide free testing for K‐12 schools (https://www.testedandprotected.org/#/).

The samples in this study were not sequenced; however, the timeframe from January to May 2021 indicates that detected infections likely did not involve any emerging variants which are likely more easily transmissible than the earlier circulating virus.^8^ As we await vaccination of younger age groups and as the dominant strain of COVID‐19 is now the more transmissible Delta variant, transmission risk remains in unvaccinated children in schools. Therefore, continued adherence to nonpharmaceutical prevention strategies is important to ensure safe school instruction.^9^

Other informal observations from the surveillance program are informative. First, we observed staff and students consistently maintaining mitigation strategies, especially among those participating in extracurricular activities who knew they were being tested weekly and did not want to jeopardize their participation. Second, with constant engagement with students and staff throughout the year, we noticed that it was easy to transition from talking about testing to vaccination. At present, 93% of staff are fully vaccinated, which was highest among public school districts in the county.

### IMPLICATIONS FOR SCHOOL HEALTH

Practices to prevent COVID‐19 transmission with return to schooling are highly controversial. Safety of children is of paramount importance and efforts should be made to prevent compromising education. New and existing partnerships between local public health, schools, and local hospital networks should be leveraged to coordinate testing, contact tracing, and vaccination to effectively reduce school transmission. Communication should be able to occur on a daily basis to provide clear and effective guidance potentially using innovative electronic platforms and engagement with the community for educational and contact tracing purposes.

This surveillance demonstrates that despite the increase of transmission in the community, schools can still safely open and remain safe spaces as long as mitigation practices are not dropped. We need to continue to take into account other changes such as vaccination uptake, new variants, and new symptom profiles. However, if a surveillance program like ours is not possible, schools should retain the basics of viral transmission prevention including masking, physical separation and cohorting, and vaccination of those who are eligible.

### Human Subjects Approval Statement

This study protocol was approved by the Case Western Reserve University Institutional Review Board (IRB#STUDY20201542).

### Conflict of Interest

The authors declare no conflict of interest.

### Author Contribution

P.R.G., H.G., R.M., M.D., J.G., and D.C. conceived and designed the work presented in the manuscript. P.R.G. analyzed the data, with P.R.G., J.R., and K.C.S. interpreting the data. P.R.G. drafted the article and all authors contributed to revising the manuscript critically for intellectual content. All authors provided final approval of the manuscript.

## Supporting information


**Figure S1.** Percent positivity of SARS‐CoV‐2 within the School Testing Program and in Lorain County from January to June 2021.
