# Supplementary material for: Back to the Basics: A COVID‐19 Surveillance Program Within a Local School District
Source: J Sch Health. 2022 Feb 23;92(5):469–73. doi: 10.1111/josh.13149 (PMC9115127; doi:10.1111/josh.13149)
Supplement: Supplementary file 1 — Figure S1. Percent positivity of SARS‐CoV‐2 within the School Testing Program and in Lorain County from January to June 2021. [file JOSH-92-469-s001.docx]

Supplemental Figure 1. Percent positivity of SARS-CoV-2 within the School Testing Program and in Lorain County from January to June 2021.
